# First line treatment selection modifies disease course and long-term clinical outcomes in *Mycobacterium avium* complex pulmonary disease

**DOI:** 10.1038/s41598-021-81025-w

**Published:** 2021-01-13

**Authors:** Kiyoharu Fukushima, Seigo Kitada, Sho Komukai, Tomoki Kuge, Takanori Matsuki, Hiroyuki Kagawa, Kazuyuki Tsujino, Mari Miki, Keisuke Miki, Hiroshi Kida

**Affiliations:** 1 Department of Respiratory Medicine, National Hospital Organization, Osaka Toneyama Medical Centre, 5-1-1 Toneyama, Toyonaka, Osaka Japan; 2 Department of Respiratory Medicine and Clinical Immunology, Osaka University Graduate School of Medicine, 2-2 Yamadaoka, Suita, Osaka Japan; 3 Department of Respiratory Medicine, Yao Tokushukai General Hospital, 1-17 Wakakusa-cho, Yao, Osaka Japan; 4 Department of Biomedical Statistics, Graduate School of Medicine, Osaka University, Osaka, Japan; 5 Institute for Open and Transdisciplinary Research Initiatives, Osaka University, Osaka, Japan

**Keywords:** Immunology, Microbiology, Health care, Medical research, Risk factors

## Abstract

The combination of rifamycin (RFP), ethambutol (EB), and macrolides is currently the standard regimen for treatment of *Mycobacterium avium* complex pulmonary disease (MAC-PD). However, poor adherence to the standardized regimens recommended by current guidelines have been reported. We undertook a single-centred retrospective cohort study to evaluate the long-term outcomes in 295 patients with MAC-PD following first line treatment with standard (RFP, EB, clarithromycin [CAM]) or alternative (EB and CAM with or without fluoroquinolones (FQs) or RFP, CAM, and FQs) regimens. In this cohort, 80.7% were treated with standard regimens and 19.3% were treated with alternative regimens. After heterogeneity was statistically corrected using propensity scores, outcomes were superior in patients treated with standard regimens. Furthermore, alternative regimens were significantly and independently associated with sputum non-conversion, treatment failure and emergence of CAM resistance. Multivariate cox regression analysis revealed that older age, male, old tuberculosis, diabetes mellitus, higher C-reactive protein, and cavity were positively associated with mortality, while higher body mass index and *M. avium* infection were negatively associated with mortality. These data suggest that, although different combination regimens are not associated with mortality, first line administration of a standard RFP + EB + macrolide regimen offers the best chance of preventing disease progression in MAC-PD patients.

## Introduction

*Mycobacterium avium* complex pulmonary disease (MAC-PD) has been increasingly implicated in a broad range of infectious conditions worldwide^1^. Multi-drug combination therapies are essential for effective treatment of MAC-PD^2^. The combination of rifamycin (RFP), ethambutol (EB), and macrolides is currently considered the standard regimen^3–5^. Although a key role of macrolides is generally well accepted^4–6^, no large studies, excepting several observational cohort studies, case series, and systematic reviews, have validated the appropriateness of standard regimens^7–9^. Hence, a lack of clear evidence may contribute to poor adherence to the standard regimens specified in current guidelines. Depletion of RFP or replacement of RFP or EB by alternatives, including fluoroquinolones (FQs), frequently occurs in clinical practice for various reasons^10,11^.

We conducted the present retrospective study, using the largest cohort and longest observation period ever reported from a clinical institution, to assess the impact of first-line treatment selection on the outcomes of MAC-PD patients. In our analyses using propensity score matching to perform unbiased comparisons, we considered various risk factors identified in previous studies including our own^12–15^. We used univariate and multivariate analyses with strict statistical thresholds to explore prognostic factors associated with treatment outcomes.

## Results

### Baseline characteristics and comparisons of standard and alternative regimens

**Figure 1:**
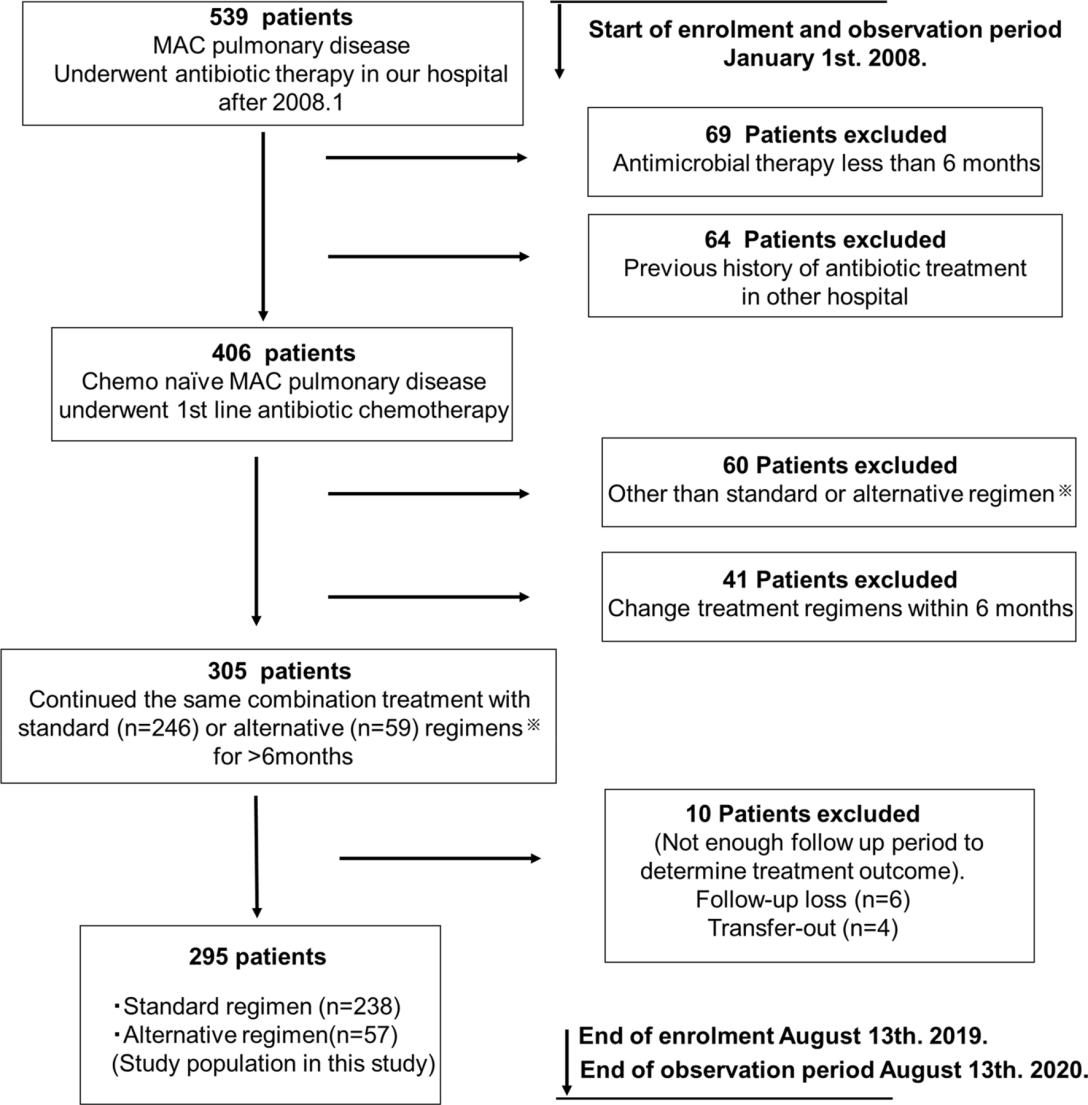
Study design. Patients were included if they were ≥ 20 years old, met the American Thoracic Society/Infectious Diseases Society of America criteria for Mycobacterium avium complex pulmonary disease (MAC-PD), no previous history of antibiotic treatment, and administration of the same combination therapy with standard or alternative regimens for 6 months or longer. Standard regimen: rifampicin (RFP) + ethambutol (EB) + clarithromycin (CAM); alternative regimen: EB + CAM ± fluoroquinolones (FQs) or RFP + CAM + FQ.

During the enrolment period, 406 chemo naïve MAC-PD patients initiated first line antibiotic therapy (Table S1). Among these patients, 295 continued treatment with standard (n = 238) or alternative (n = 57) regimens for 6 months or longer (Fig. 1 and Table S2). Factors influencing the selection of alternative regimens included: avoidance of RFP because of liver (3/57, 5.3%), thyroid (1/57, 1.8%) or digestive (1/57, 1.8%) disease, avoidance of RFP because of drug interactions (5/57, 8.8%), avoidance of EB because of ophthalmological disease (3/57, 5.3%), previous occasional prescription of FQs with acceptable tolerance or suspicion of concomitant bacterial infection (9/57, 15.8%), older age (5/57, 8.8%), low body weight (3/57, 5.3%), avoidance of three drug regimens at the patient's request owing to concerns regarding side effects (6/57, 10.5%), uncontrolled DM (2/57, 3.6%), and unclear (19/57, 33.3%) (Table S3). Parenteral aminoglycoside antibiotics were used in 85 patients. Old tuberculosis (Old Tb) was more frequent (p = 0.0373) and C-reactive protein (CRP) were higher (p = 0.0004) in the alternative regimen group (Table 1). Sputum conversion rates were higher (81.9% vs 60.0%, p = 0.0008) and rates of treatment failure were lower in the standard regimen group (31.9% vs 54.4%, p = 0.0015) (Table 1). To make unbiased comparisons between standard and alternative regimens, we further assessed patient outcomes after 1:1 statistical matching using propensity scores. This process statistically corrected for heterogeneity of patient background and balanced potential confounding factors including sex, age, body mass index (BMI), comorbidities, acid fast bacilli (AFB) stain positivity, CRP, cavity, type of disease [nodular bronchiectatic (NB) or fibrocavitary (FC)], mycobacterial species, and aminoglycoside use. To further reduce potential biases, attending pulmonary physicians were also balanced (Table S4). Forty eight patients in each group were matched for analysis (nine patients receiving alternative regimens remained unmatched). Among matched patients receiving standard regimens, 38/48 (79.2%) achieved sputum culture conversion compared with 29/48 (60.4%) of matched patients receiving alternative regimens (p = 0.0454) (Table 1). Treatment failure was significantly more frequent in matched patients receiving alternative regimens (33.3% vs. 54.2%, p = 0.0396).

**Table 1:** Baseline characteristics and treatment outcomes of MAC-PD patients.

	Total (n = 295)	Standard regimen vs alternative regimen			Matched standard regimen vs matched alternative regimen		
					Statistical matching		
		Standard regimen (n = 238)	Alternative regimen (n = 57)	p-value	Matched Standard regimen (n = 48)	Matched Alternative regimen (n = 48)	p-value
**Characteristic**							
Sex, male	70 (23.7)	53 (22.3)	17 (29.8)	0.2284	14 (29.2)	14 (29.2)	> 0.9999
Age, years	65.9 (± 10.5)	65.7(± 10.2)	66.9 (11.4)	0.4390	68.6 (± 9.5)	66.5 (± 12.1)	0.3553
BMI	18.7 (± 2.7)	18.6 (2.7)	18.7 (2.6)	0.7639	18.0 (± 2.7)	18.8 (± 2.8)	0.1314
Underlying disease							
COPD	15 (5.1)	10 (4.2)	5 (8.8)	0.1583	4 (8.3)	4 (8.3)	> 0.9999
Old Tb	15 (5.1)	9 (3.8)	6 (10.5)	0.0373*	5 (10.4)	5 (10.4)	> 0.9999
DM	27 (9.2)	22 (9.2)	5 (8.8)	0.9117	7 (14.6)	4 (8.3)	0.5235
CPA	3 (1.0)	2 (0.8)	1 (0.4)	0.4762	1 (2.1)	1 (2.1)	> 0.9999
CRP	1.3 (± 2.2)	1.1 (± 2.0)	2.2 (± 2.9)	0.0004*	2.2 (± 2.9)	2.1 (± 2.8)	0.8930
NB form	249 (84.4)	200 (84.0)	49 (86.0)	0.7181	41 (85.4)	40 (83.3)	0.7786
Cavity	127 (43.1)	103 (43.3)	24 (42.1)	0.8725	22 (45.8)	21 (43.8)	0.8374
*M .avium*	160 (54.2)	125 (53.0)	35 (61.4)	0.2266	32 (66.7)	31 (64.6)	0.8299
AFB positive	123 (41.7)	93 (39.1)	30 (52.6)	0.0623	26 (54.2)	26 (54.2)	> 0.9999
**Antibiotics treatments**							
RFP	260 (88.1)	238 (100.0)	22 (38.6)		52 (100.0)	19 (39.6)	
EB	273 (92.5)	238 (100.0)	35 (61.4)		52 (100.0)	29 (60.4)	
CAM	295 (100.0)	238 (100.0)	57 (100.0)		52 (100.0)	48 (100.0)	
LVFX	16 (5.4)	0 (0.0)	16 (28.1)		0 (0.0)	14 (29.2)	
MFLX	11 (3.7)	0 (0.0)	11 (19.3)		0 (0.0)	9 (18.8)	
GRNX	2 (0.7)	0 (0.0)	2 (3.5)		0 (0.0)	2 (4.2)	
STFX	7 (2.4)	0 (0.0)	7 (12.3)		0 (0.0)	6 (12.5)	
Aminoglycoside use	85 (28.8)	72 (30.5)	13 (22.8)	0.2649	15 (31.3)	13 (27.1)	0.6534
**Outcomes**							
Treatment duration, months	29.6 (± 22.5)	28.4 (± 21.1)	34.6 (27.1)	0.0645	26.5 (± 20.9)	32.6 (± 24.6)	0.1900
Sputum conversion	230 (78.0)	195 (81.9)	35 (60.0)	0.0008*	38 (31.3)	29 (60.4)	0.0454*
Treatment failure	107 (36.3)	76 (31.9)	31 (54.4)	0.0015*	16 (33.3)	26 (33.3)	0.0396*
Recurrence	42 (14.2)	37 (15.5)	5 (8.8)	0.1886	10 (20.8)	5 (10.4)	0.1599

Data represent n (%) or median (interquartile range).

*MAC-PD* *Mycobacterium avium* complex pulmonary disease; *BMI* body mass index; *COPD* chronic obstructive pulmonary disease; *Tb* tuberculosis; *DM* diabetes mellitus; *CPA* chronic pulmonary aspergillosis; *CRP* C-reactive protein; *NB* nodular bronchiectasis; *CAM* clarithromycin; *RFP* rifampicin; *EB* ethambutol; *LVFX* levofloxacin; *MFLX* moxifloxacin; *STFX* sitafloxacin; *GRNX* garenoxacin.

### Risk factors for sputum non-conversion

In univariate analyses, high CRP levels (p = 0.0008), cavity (p = 0.0208), AFB stain positivity (p = 0.0105), and alternative regimens (p = 0.0027) were significantly associated with failure of sputum conversion (Table 2). Multivariate Cox regression analysis revealed that cavity (p = 0.0333) and alternative regimens (p = 0.0311) were independently associated with failure of sputum conversion (Table 2).

**Table 2:** Predictors of sputum culture conversion in patients with MAC-PD.

	Conversion (n = 230)	Non-conversion (n = 65)	Univariate analysis		Multivariate analysis	
			p-value	HR (95%CI)	p-value	Adjusted HR (95%CI)
**Characteristic**						
Sex, male	50 (21.7)	20 (30.8)	0.3451	1.16 (0.86–1.60)		
Age, years	65.6 (± 10.4)	66.9 (± 10.8)	0.7169	1.00 (0.98–1.01)		
BMI	18.9 (± 2.6)	18.1 (± 2.8)	0.3037	1.00 (1.00–1.01)		
Underlying disease						
COPD	11 (4.8)	4 (6.2)	0.8199	0.94 (0.49–1.60)		
Old Tb	9 (3.9)	6 (9.2)	0.265	0.71 (0.35–1.27)		
DM	18 (7.8)	9 (13.8)	0.2959	0.78 (0.47–1.23)		
CPA	2 (0.9)	1 (1.5)	0.9555	0.96 (0.16–3.00)		
CRP	1.0 (± 2.0)	2.2 (± 2.6)	0.0008*	0.89 (0.82–0.96)	0.0646	0.93 (0.86–1.00)
NB form	191 (83.0)	58 (89.2)	0.3618	1.18 (0.82–1.64)	0.1255	1.37 (0.91–2.03)
Cavitation	89 (38.7)	38 (58.5)	0.0208*	0.73 (0.56–0.95)	0.0333*	0.72 (0.52–0.97)
*M. avium*	128 (55.7)	32 (49.2)	0.5951	1.07 (0.83–1.39)		
AFB stain positive	86 (37.4)	37 (56.9)	0.0105*	0.71 (0.54–0.92)	0.0583	0.77 (0.58–1.01)
Standard regimen	195 (84.8)	43 (66.2)	Ref	1.00		
Alternative regimen	36 (15.7)	21 (32.3)	0.0027*	0.59 (0.41–0.84)	0.0311*	0.68 (0.46–0.97)
Aminoglycoside use	70 (30.4)	15 (23.1)	0.5856	1.08 (0.81–1.43)		
Adjuvant surgery	10 (4.3)	0 (0.0)	0.1279	1.76 (0.83–3.23)	0.1797	1.66 (0.77–3.17)

Data represent n (%) or median (interquartile range).

*MAC-PD* *Mycobacterium avium* complex pulmonary disease; *HR* hazard ratio; *CI* confidence interval; *BMI* body mass index; *COPD* chronic obstructive pulmonary disease; *Tb* tuberculosis; *DM* diabetes mellitus; *CRP* C-reactive protein; *CPA* chronic pulmonary aspergillosis; *NB* nodular bronchiectasis; *AFB* acid-fast bacilli.

### Risk factors for treatment failure

Among the 295 patients, 107 (36.3%) experienced treatment failures (Table S5). In univariate analyses, treatment failure was significantly associated with low BMI (p = 0.0197), high CRP levels (p = 0.0253), cavity (p = 0.0064), AFB stain positivity (p = 0.0024), and alternative regimens (p = 0.0018) were positively associated with treatment failure, while adjuvant surgery (p = 0.0003) was negatively associated with treatment failure. Multiple logistic regression analysis revealed that AFB stain positivity (p = 0.021), cavity (p = 0.0136), and alternative regimens (p = 0.017) were independently and significantly associated with treatment failure. Although not statistically significant, aminoglycoside use was less frequent in the treatment failure group. Successful completion was achieved in 188 patients. We did not identify any risk factors for recurrence in univariate analyses (Table S6). The non-cavitary NB form of the disease was more frequent in the recurrent disease group, but this difference was not statistically significant.

### Risk factors for development of CAM resistance

All patients were tested for drug susceptibility prior to treatment. Susceptibility to CAM was classified as sensitive (MIC ≤ 4 µg/mL) in 294 (99.6%) patients and resistant (MIC ≥ 32 µg/mL) in 1 patient (0.4%). Acquired CAM resistance increased over the observation period. Over the observation period, CAM resistance was detected in 36 (12.2%) patients (standard regimens, n = 22 [9.2%]; alternative regimens, n = 14 [24.6%]). In univariate analyses, emergence of CAM resistance was associated with BMI (p < 0.0001), chronic obstructive pulmonary disease (COPD) (p = 0.0047), longer treatment duration (p < 0.0001), and alternative regimens (p = 0.0019) (Table 3). Multiple logistic regression analysis revealed that COPD (p = 0.0226), longer treatment duration (p = 0.0046), and alternative regimens (p = 0.0079) were significantly and independently associated with CAM resistance (Table 3). A comparison of susceptibility to FQs is shown in Table S7. Among FQs, the minimum inhibitory concentrations (MICs) of sitafloxacin (STFX) were lowest and, the MICs of levofloxacin (LVFX) were generally higher than those of moxifloxacin (MFLX).

**Table 3:** Risk factors for development of CAM resistance in MAC-PD patients.

	CAM-resistance Not detected (n = 259)	CAM-resistance detected (n = 36)	Univariate analysis		Multivariate analysis	
			p-value	OR (95%CI)	p-value	Adjusted OR (95%CI)
**Characteristic**						
Sex, male	56 (21.6)	14 (38.9)	0.0296*	2.31 (1.11–4.80)		
Age, years	65.9 (± 10.3)	66.1 (± 12.0)	0.923	1.00 (0.97–1.04)		
BMI	18.7 (2.6)	18.5 (3.2)	0.3903	0.97 (0.86–1.10)	< .0001*	0.91 (0.87–0.95)
Underlying disease						
COPD	9 (3.5)	6 (16.7)	0.0047*	5.56 (1.85–16.69)	0.0226*	4.32 (1.22–15.19)
Old Tb	12 (4.6)	3 (8.3)	0.3777	1.87 (0.50–6.98)		
DM	25 (9.7)	2 (5.6)	0.4308	0.55 (0.12–2.43)		
CPA	3 (1.2)	0 (0.0)	0.9909			
CRP	1.2 (± 2.1)	2.0 (2.8)	0.071	1.13 (0.996–1.29)		
NB form	219 (84.6)	30 (83.3)	0.8498	1.10 (0.43–2.80)		
Cavity	104 (40.2)	23 (63.9)	0.0004*	2.31 (1.45–3.69)	0.0575	2.10 (0.98–4.53)
*M. avium*	139 (53.7)	21 (58.3)	0.5989	1.21 (0.60–2.45)		
AFB stain positive	104 (38.6)	19 (52.8)	0.0751	1.89 (0.94–3.82)		
Treatment duration, months	28.0 (± 21.1)	41.0 (28.9)	< .0001*	1.03 (1.02–1.04)	0.0046*	1.02 (1.01–1.04)
Standard regimen	216 (83.4)	22 (61.1)	Ref	1.00		
Alternative regimen	43 (16.6)	14 (38.9)	0.0019*	2.54 (1.41–4.58)	0.0079*	2.96 (1.33–6.60)
Aminoglycoside use	77 (29.7)	8 (22.2)	0.3537	0.68 (0.29–1.55)		

Data represent n (%) or median (interquartile range).

Drug susceptibility tests were re-examined after treatment initiation in 53.4% (127/238) of patients receiving standard regimens and 62.5% (35/57) of patients receiving alternative regimens.

*MAC-PD* *Mycobacterium avium* complex pulmonary disease; *OR* odds ratio; *CI* confidence interval; *BMI* body mass index; *COPD* chronic obstructive pulmonary disease; *Tb* tuberculosis; *DM* diabetes mellitus; *CRP* C-reactive protein; *NB* nodular bronchiectasis; *CAM* clarithromycin.

### Risk factors for all-cause and MAC-PD-associated mortality

**Figure 2:**
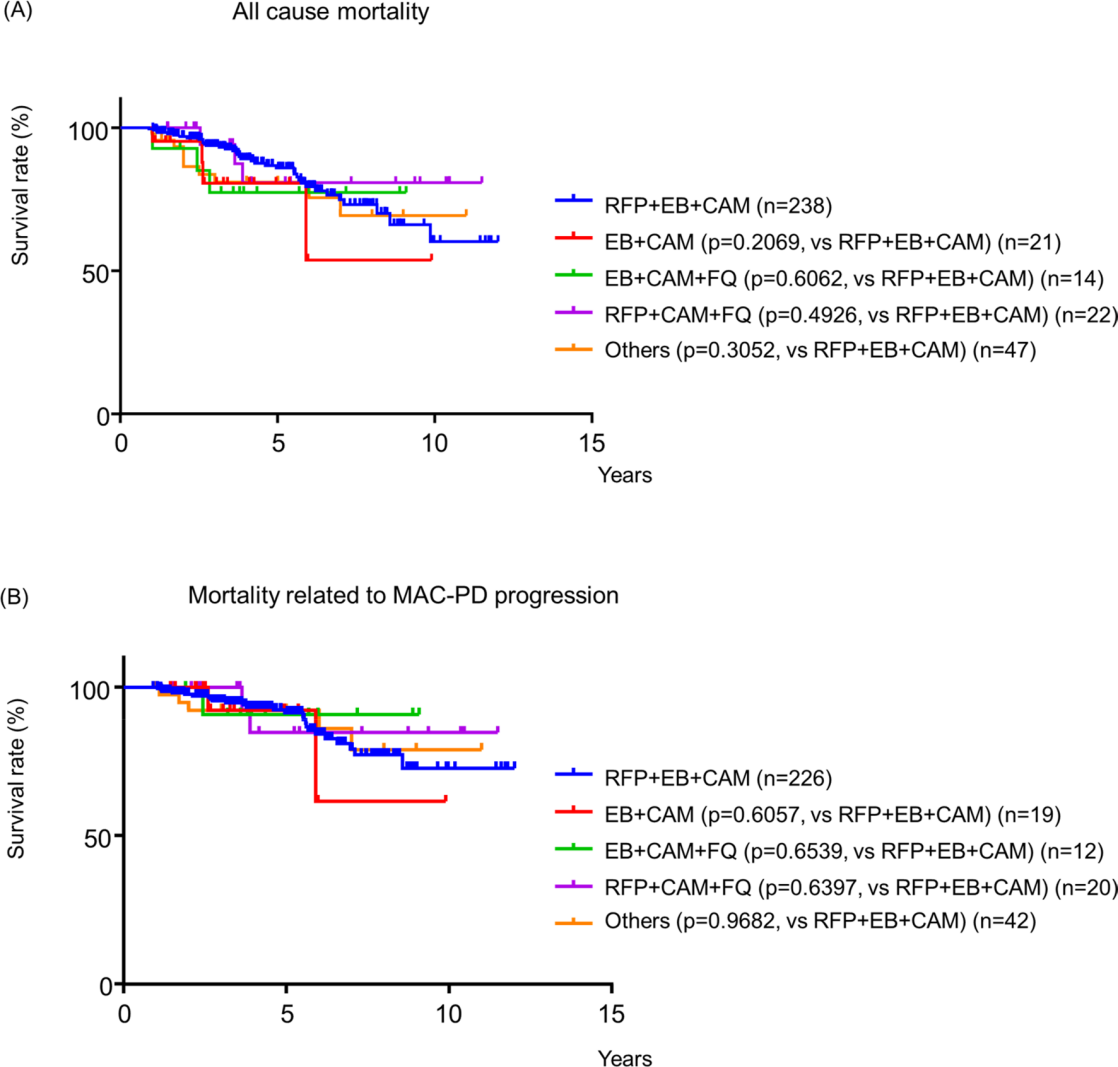
Survival curves for different 1st line combination regimen. (**A**) All cause mortality, (**B**) Mortality from MAC-PD progression. Survival curves were analyzed by log rank test.

Deaths from any cause occurred in 48 patients. Among these patients, 30 died from MAC-PD progression, five died from pneumonia, three died from fungal infection, and 10 died from other causes (Fig. S1). We analysed the prognostic factors associated with all-cause mortality (Table 4). Multivariate Cox regression analysis revealed that male sex (p < 0.0001), older age (p = 0.0315), BMI (p < 0.0001), Old Tb (p = 0.0247), DM (p = 0.0188), cavity (p = 0.0472) and CRP level (p < 0.0001) were positively associated with mortality, while M. *avium* infection (p = 0.0142) was negatively associated with mortality. Chronic pulmonary aspergillosis (CPA) was diagnosed in 23 (7.8%) patients during the follow-up period. Although pre-existing CPA was borderline significant in multivariate analysis of all-cause mortality (p = 0.0502), MAC-PD patients who developed CPA showed extremely poor prognoses (Fig. S2). Multiple logistic regression analysis revealed that higher CRP was significantly and independently associated with development of CPA (Table S8). We further analysed prognostic factors for mortality related to MAC-PD progression (Table S9). Multivariate Cox regression analysis revealed that male sex (p = 0.0075), BMI (p = 0.0003), Old Tb (p = 0.0409), CRP level (p = 0.0023), and cavity (p = 0.0015) were positively associated with mortality, while *M. avium* infection (p = 0.0081) was negatively associated with mortality. In univariate and multivariate Cox regression analysis, choice of standard or alternative regimens was not associated with all-cause mortality or mortality related to MAC-PD progression. We also analysed associations between first-line combination regimens and mortality by log rank tests of survival curves. In this analysis, we also evaluated patients treated with regimens other than the standard and alternative regimens. Among 60 patients treated with other regimens (Fig. 1, Table S1), 47 received the same regimen for 6 months or longer and accrued sufficient observation time to assess outcomes. Survival curves of all-cause mortality and mortality from MAC-PD progression were comparable for patients treated with all regimens (standard, alternative or other) (Fig. 2).

**Table 4:** Risk factors for MAC-PD mortality.

	Censored (n = 247)	Death (n = 48)	Univariate analysis		Multivariate analysis	
			p-value	HR (95%CI)	p-value	Adjusted HR (95%CI)
**Characteristic**						
Sex, male	49 (11.7)	21 (44.7)	0.0002*	3.19 (1.77–5.71)	< .0001*	3.00 (1.50–5.92)
Age, years	65.3 (± 10.6)	69.1 (± 9.4)	0.0008*	1.06 (1.02–1.10)	0.0315*	1.04 (1.00–1.08)
BMI	18.9 (± 2.6)	17.4 (± 2.9)	< .0001*	0.76 (0.66–0.87)	< .0001*	0.80 (0.70–0.91)
Underlying disease						
COPD	7 (2.8)	8 (17.0)	0.0046*	3.56 (1.54–7.23)	0.2279	1.88 (0.66–4.97)
Old Tb	7 (2.8)	8 (17.0)	0.003*	3.83 (1.66–7.80)	0.0247*	3.10 (1.15–9.12)
DM	16 (6.5)	11 (23.4)	0.0005*	4.02 (1.94–7.70)	0.0188*	2.84 (1.20–6.40)
CPA	1 (0.4)	2 (4.3)	0.0072*	16.3 (2.60–56.1)	0.0502	7.32 (0.998–35.55)
CRP	1.1 (± 2.1)	2.4 (± 2.5)	< .0001*	1.23 (1.13–1.33)	< .0001*	1.22 (1.06–1.41)
NB form	212 (85.5)	37 (78.7)	0.1846	0.61 (0.31–1.29)		
Cavity	92 (37.2)	35 (72.9)	< .0001*	4.24 (2.27–8.45)	0.0472*	2.11 (1.01–4.56)
*M. avium*	143 (57.7)	17 (36.2)	0.0028*	0.41 (0.22–0.74)	0.0106*	0.44 (0.23–0.83)
AFB stain positive	94 (37.9)	29 (61.7)	0.0123*	2.09 (1.17–3.84)		
Standard regimen	201 (81.0)	37 (78.7)	Ref	1.00		
Alternative regimen	47 (19.0)	10 (21.3)	0.7212	1.68 (0.88–3.46)		
Aminoglycoside use	74 (29.8)	11 (23.4)	0.1173	0.60 (0.29–1.13)		
Adjuvant surgery	10 (4.0)	0 (0.0)	0.0822		0.0793	

Data represent n (%) or median (interquartile range).

*MAC-PD* *Mycobacterium avium* complex pulmonary disease; *HR* hazard ratio; *CI* confidence interval; *BMI* body mass index; *COPD* chronic obstructive pulmonary disease; *Tb* tuberculosis; DM, diabetes mellitus; *CRP* C-reactive protein; *NB* nodular bronchiectasis; *CPA* chronic pulmonary aspergillosis.

## Discussion

The results of our study clearly showed that standard RFP + EB + CAM regimens should be considered as first-line treatments. Standard regimens offer patients the best chance for sputum conversion, to prevent treatment failure, and to avoid emergence of CAM resistance.

Long-term outcomes following different combination therapies for MAC-PD are poorly understood, and this lack of clear evidence might contribute to poor adherence to the standard regimens recommended by current guidelines^10,11^. In daily practice, some physicians, especially those treating populations with a low prevalence of tuberculosis, may consider it easier to use FQs than RFP or EB. Other physicians may have experienced the serious side effects caused by RFP or EB in patients under their care. However, we believe that one of the most important reasons for poor adherence to standard regimens is that the superiority of such regimens is not adequately recognised. No large studies have validated the appropriateness of current treatment guidelines for MAC-PD^2,16^. Therefore, to provide clearer evidence, we conducted this retrospective study to compare the effectiveness of different treatment approaches using a large cohort, long observation times, and rigorous statistical analyses.

We previously conducted a retrospective study of highly progressive MAC-PD patients. We assessed the effects of standard regimens and incorporation of aminoglycosides and demonstrated the prognostic importance of DM and CPA in cavitary MAC-PD^12^. Our previous study included 125 progressive MAC-PD patients hospitalized for induction of combination antimicrobial drug therapy; this was a heterogeneous population with severe or advanced disease. Therefore, to gain broader applicability of these findings, we enrolled all chemo-naive MAC-PD patients receiving first line antimicrobial therapies for 6 months or longer (standard or alternative regimens). To reduce heterogeneity and potential bias, we excluded patients treated with regimens that had already proven ineffective or even harmful because of the onset of macrolide resistance (EB + FQ, CAM + FQ, RFP + CAM, CAM alone, and RFP + EB). We also excluded patients who changed treatment regimens within 6 months of treatment initiation. Regimen changes were mostly occurred because of the onset of drug side effects. Assessing the effects of treatment cross-over and drug side effects requiring regimen change on treatment outcomes is a major challenge. Therefore, we analysed only patients who continued the same combination treatment with standard or alternative multidrug regimens for 6 months or longer.

FQs were reported to be effective against MAC-PD in vitro and in vivo^17,18^. However, data on the use of FQs in combination therapy regimens are lacking^19,20^. Furthermore, concerns have been raised regarding the use of FQs for treatment of MAC-PD. Specifically, application of Clinical Laboratory and Standards Institute breakpoints in clinical practice has shown little activity of FQs^21,22^, and there is potentially mild antagonism between FQs and CAM in combination therapies^17^. Moreover, FQs have received warnings from the US Food and Drug Administration and the European Medicines Agency for significant adverse effects, including peripheral neuropathy, psychosis, hypoglycaemia, tendon rupture, and aortic dissection^23^. Recently, Khadawardi et al.^24^ reported that substitution of RFP or EB with FQs resulted in similar treatment outcomes compared with standard triple-drug regimens. However, their study population included patients who crossed over to FQ-containing regimens because of the onset of drug side effects; such patients were excluded from our study. Furthermore, a population-based study showed no difference in mortality associated with different combination regimens^25^. However, both were retrospective studies of heterogeneous populations, and various confounding factors were not statistically controlled. In contrast, we compared clinical outcomes between standard regimens and alternative regimens after matching for various potential confounding factors using propensity scores.

In this study, we showed for the first time that alternative regimens were independent risk factors for acquisition of CAM resistance in patients with MAC-PD. The association between CAM resistance and alternative regimens may be related to the fact that 38.6% of patients who avoided standard regimens did not receive EB. Griffith et al.^26^ reported that administration of macrolides in the absence of EB was a risk factor for development of macrolide resistance. Whether RFP has additional effects in protecting against CAM resistance is not yet fully understood. We speculate that RFP and EB may be required for prevention of CAM resistance through augmentation of anti-mycobacterial clearance or unknown mechanisms of interplay between these drugs^4^. No patients in our study who started treatment with alternative regimens were ever prescribed standard regimens by their attending physicians, even if treatment failed. Therefore, avoidance of standard regimens and selection of alternative regimens as first line treatments may lead to loss of opportunity to use first-line drugs, which might in turn contribute to the high frequency of CAM resistance development in patients treated with alternative regimen.

Prognostic factors for MAC-PD have been identified in many retrospective studies^13–15^. In our study, male sex, age, low BMI, Old Tb, DM, cavity, higher CRP, and mycobacterial species were independently associated with mortality. For the first time, we showed that *M. avium* infection was significantly and independently associated with reduced all-cause mortality and mortality related to MAC-PD progression in progressive MAC-PD patients. This finding coincides with those of a previous report showing worse prognoses for patients with M. *intracellulare* lung disease^27^. In our study, choice of combination regimens was not associated with mortality of all-cause and mortality from MAC-PD progression; instead, mortality was mostly associated with host immunological status, disease activity, and comorbidities.

We also found that MAC-PD patients with CPA had unfavourable outcomes. Azoles such as voriconazole and itraconazole are important for the treatment of CPA. However, treatment with azoles necessitates avoidance of RFP because of drug interactions. Indeed, concerns regarding drug interactions have led to inappropriate treatment of both MAC-PD and CPA. In our study, nine patients were diagnosed with CPA during first-line MAC-PD therapy. This might have contributed to the extremely poor prognoses of MAC-PD patients who developed CPA. CPA mostly occurs in patients with cavitary non-NB disease, and higher CRP was an independent risk factor for the development of CPA. Hence, immunological processes might be associated with poor outcomes in MAC-PD patients with CPA. Cavity formation during mycobacterial infection is associated with massive necrosis and loss of normal architecture in peripheral areas of the lung, and is induced by macrophages and activated T cells responding to infection as well as by delayed-type hypersensitivity to mycobacterial antigens^28^. This may also be the case for MAC-PD. CPA can lead to host hypersensitivity, which may contribute to destruction of lung tissue in combination with inflammatory responses to *Aspergillus* organisms^29^. Therefore, co-infection with MAC and *Aspergillus* species may exaggerate lung tissue destruction and subsequent respiratory failure.

Our study had several limitations. First, we could not exclude potential confounding factors (e.g., microbiological and other laboratory features) because of the retrospective nature of the study. Second, as a retrospective study, attending pulmonary physicians made decisions independently regarding timing and selection of antimicrobial therapies. Therefore, physician bias could have potentially influenced prognosis. To reduce potential biases, we took attending physician behaviour into account in propensity score matching. Furthermore, we thoroughly and carefully examined the reasons why standard regimens were not initiated in patients receiving alternative regimens and clarified the factors that influencing choice of regimens. Finally, among patients treated with FQ-containing three drug alternative regimens, only 38.9% received EB and 44.4% received LVFX. This may have reduced the effectiveness of FQ-containing regimens and affected our results. Combined administration of CAM and LVFX produced unfavourable clinical outcomes in patients with MAC-PD^30^, although multiple studies have demonstrated modest activities of FQ-containing regimens, irrespective of the FQ used^17,19,20,24,31^. Indeed, in our study, the MICs of LVFX were generally higher than those of other FQs such as MFLX and STFX (Table S7). Frequent use of LVFX may be related to the health insurance system in Japan: LVFX is the only FQ covered by health insurance for pulmonary tuberculosis therapy, and therefore administration of LVFX to MAC-PD patients is easier for clinicians than administration of other FQs.

In conclusion, first line administration of standard RFP + EB + CAM regimens offers the best chance of preventing disease progression in MAC-PD patients. First-line use of alternative regimens such as FQ-containing regimens without clear justification should be avoided. Alternative regimens should be used only in selected patients or set aside for second- or third-line treatment.

## Methods

### Study design and patients

This retrospective study was performed at the National Hospital Organization, Osaka Toneyama Medical Centre, a referral centre for respiratory diseases with 410 inpatient beds. The enrolement period was from 1 January 2008 to 13 August 2019 (Fig. 1). The medical records of patients with MAC-PD were reviewed, and patients were included if they were ≥ 20 years old, met the American Thoracic Society/Infectious Diseases Society of America criteria for MAC-PD^3^, started first line antibiotic therapy for MAC-PD between 1 January 2008 and 13 August 2019, and continued the same combination therapy with standard or alternative multidrug regimens for 6 months or longer. We defined RFP + EB + CAM regimens as standard regimens and RFP + CAM + FQ or EB + CAM + /− FQ regimens as alternative regimens. Patients who received previous combination antibiotic treatments for MAC-PD or patients who had received prior antibiotic therapy including CAM monotherapy for more than 1 month were excluded. Patients lost to follow up (including patients with insufficient follow up time to determine treatment outcome and transferred out to another hospital) were also excluded. Chemo naïve MAC-PD patients were defined as MAC-PD patients who had not been received previous combination antibiotic treatments and any antibiotic therapy for more than 1 month. Only two patients with NB type MAC-PD received intermittent therapy with standard regimens; these patients were also excluded. None of the patients tested positive for human immunodeficiency virus. All patients were followed until their last visit, death, or the end of the observation period (13 August 2020).

### Data collection

Baseline clinical parameters, including age, sex, BMI, smoking status, and comorbidities, were obtained before treatment initiation. Treatment duration, antimicrobial treatments for MAC-PD, bacterial culture results, and chest computed tomography (CT) findings were obtained from medical records. Diagnosis of chronic pulmonary aspergillosis was defined by compatible clinical symptoms, compatible radiological findings, positive *Aspergillus* serology [positive *Aspergillus* IgG (Bio-Rad, Hercules, CA, USA), positive precipitin tests (Microgen Bioproducts Ltd., Camberley, Surrey, UK), or strongly positive *Aspergillus* antigen (Bio-Rad)], or isolation of *Aspergillus* species from respiratory samples. These definitions were consistent with the current European guidelines for CPA^32^. Indications for adjuvant lung surgery were discussed at the time of diagnosis or 3–6 months after multidrug treatment at a weekly multidisciplinary meeting of pulmonary physicians and surgeons. Consideration was given to postoperative cardiopulmonary function. Surgical resection was performed in the following cases: (1) MAC-PD refractory to multiple drug therapy; (2) cavitary lesions and/or severe bronchiectasis; or (3) development of complications such as massive haemoptysis.

### Radiological evaluation

Radiographic abnormalities were classified according to distinct patterns observed on chest CT^15^. Patients with fibrocavitary lesions and pleural thickening mainly in the upper lobes on CT were diagnosed with FC disease. Patients with multiple nodules on CT and bronchiectasis were diagnosed with NB disease. Patients with no specific pattern on CT, including solitary pulmonary nodules, were diagnosed with unclassifiable disease. Chest CT findings were assessed by two pulmonologists blinded to the clinical data. Because cavities were frequently accompanied by bronchiectasis and bronchiolitis in MAC pulmonary disease, we carefully distinguished between cavities and bronchiectasis. Discrepancies were resolved through a consensus review by pulmonary physicians and a pulmonary radiologist.

### Antibiotic therapy

FQs used in this study included STFX, MFLX, garenoxacin, and LVFX^19^. Aminoglycoside antibiotics were administered intramuscularly (kanamycin, streptomycin) or intravenously (amikacin). Streptomycin and kanamycin were administered three times a week for the first several months at the discretion of the attending physician. Amikacin was administered daily for 28 days, followed by kanamycin or streptomycin for the first several months. Treatment regimens were evaluated 3 months after treatment initiation. Drugs were dosed as follows: RFP, 450–600 mg/day or 10 mg/kg/day; EB, 750 mg/day or 15 mg/kg/day; CAM, 600–800 mg/day; STFX, 200 mg/day; MFLX, 400 mg/day; garenoxacin, 400 mg/day; LVFX, 500 mg/day; kanamycin, streptomycin, and amikacin, 10–15 mg/kg.

### Sputum examination

Sputum cultures were assessed for AFB using 2% Ogawa egg medium (Japan BCG, Tokyo, Japan) or mycobacterial growth indicator tubes (Becton Dickinson, Tokyo, Japan). Non-tuberculous mycobacterial species were identified using the AccuProbe (Gen-Probe Inc., San Diego, CA, USA) or COBAS AMPLICOR (Roche Diagnostics, Tokyo, Japan) systems or using DNA–DNA hybridization assays (Kyokuto Pharmaceutical Industrial, Tokyo, Japan). CAM susceptibility was determined by broth microdilution (BrothMIC NTM; Kyokuto Pharmaceutical Industrial). CAM resistance was defined as MIC ≥ 32 µg/mL^33^. Susceptibility tests to CAM were performed before treatment initiation and at the time of suspected recurrence or development of refractory disease.

### Definitions of sputum conversion, treatment failure, recurrence, and treatment success

At the end of follow-up, patient status was recorded in terms of deceased or alive, microbiological or radiological recurrence, and cause of death if applicable. Sputum examinations on Ogawa solid medium were performed at 1 to 3-month intervals following treatment initiation. Sputum conversion was defined as more than three consecutive negative sputum cultures over a period of 3 months^34^. Treatment failure was defined as re-emergence of multiple positive cultures or persistence of positive respiratory cultures following > 12 months of antimycobacterial therapy while the patient remained on treatment. Recurrence was defined as re-emergence of at least two positive respiratory sample cultures after cessation of antimycobacterial treatment. Successful completion was defined as treatment cessation after sustained negative sputum conversion or sustained negative sputum conversion for more than 12 months.

### Statistical analysis

All statistical analyses were performed using GraphPad Prism version 7 (GraphPad Software, San Diego, CA, USA) and JMP Pro 13 (SAS Institute, Gary, NC, USA). Continuous variables were reported as means and standard deviations. Differences between continuous variables were assessed using the t-test and differences between categorical variables were assessed using the χ^2^ test. When any cell had an expected count of less than 5, Fisher's exact test was used instead of the χ^2^ test. To assess potential risk factors, univariate and multivariate analyses with logistic and Cox regression models were used. We conducted estimation and variable selection by linear regression with the Lasso-type penalty. A 1:1 propensity score matching of age, sex, BMI, CRP, comorbidities (COPD, Old Tb lesions, DM, cavity, AFB stain positivity, type of disease [NB or FC], mycobacterial species, aminoglycoside use, and attending pulmonary physician) was performed for patients receiving standard and alternative regimens. For matching by the nearest neighbour method using the Mahalanobis distance, a calliper of 0.20 and a random number seed value of 111 were used. Values of p < 0.05 were considered statistically significant.

### Use of human participants

The experimental protocol for data involving human participants followed the Ethical Guidelines of the Japan Ministries of Health and Labour for Medical and Health Research Involving Human Subjects. All experiments were conducted in accordance with the principles laid out in the Declaration of Helsinki. The study was approved by the Institutional Review Board of the National Hospital Organization, Osaka Toneyama Medical Centre (TNH-2019063). The IRB committee waived the requirement for informed consent for a retrospective review of participant data. The opt-out recruitment method was applied to provide an opportunity to decline participation for all patients. Members of the IRB committee were as follows; Harutoshi Fujimura, Tsuyoshi Matsumura, Yukiyasu Takeuchi, Masahide Mori, Toshihiko Yamaguchi, Keisuke Miki, Hiroyuki Ueno, Makiko Sawamoto, Taku Shiomi, Motomu Shimoda, Hironori Tsukada, and Minako Nakano.

## Supplementary Information

Supplementary Information 1.

Supplementary Information 2.

## Data Availability

The datasets supporting the conclusions of this article are included within the article and its additional supplemental files.
