# Supplementary material for: First line treatment selection modifies disease course and long-term clinical outcomes in Mycobacterium avium complex pulmonary disease
Source: Sci Rep. 2021 Jan 13;11:1178. doi: 10.1038/s41598-021-81025-w (PMC7807086; doi:10.1038/s41598-021-81025-w)

Figure S1. Analysis of causes of deaths.

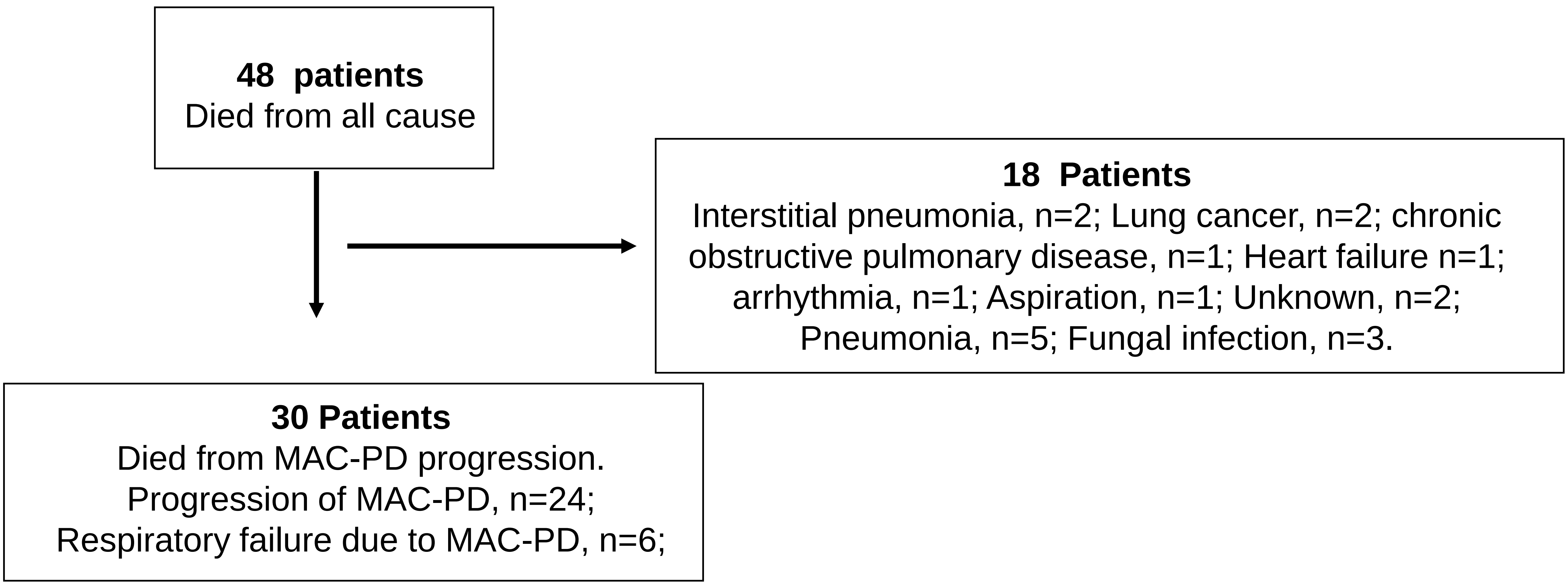

Figure S2. Survival curves of Mycobacterium avium complex pulmonary disease (MAC-PD) in patients who did or did not develop chronic pulmonary aspergillosis (CPA). P value was calculated by log-rank test.

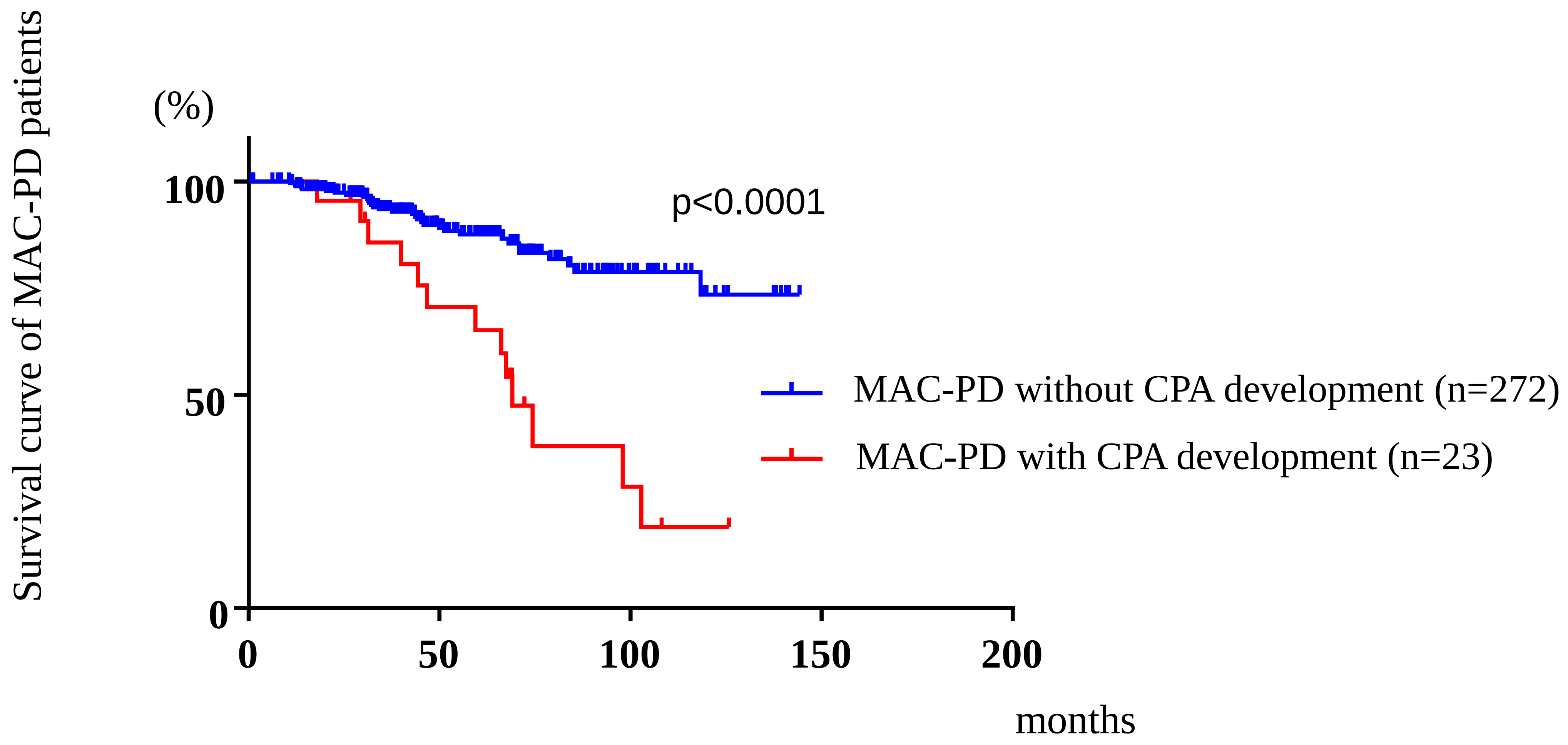

Supplement: Supplementary file 1 — Supplementary Information 1. [file 41598_2021_81025_MOESM1_ESM.pdf]
