# Supplementary material for: First line treatment selection modifies disease course and long-term clinical outcomes in Mycobacterium avium complex pulmonary disease
Source: Sci Rep. 2021 Jan 13;11:1178. doi: 10.1038/s41598-021-81025-w (PMC7807086; doi:10.1038/s41598-021-81025-w)
Supplement: Supplementary file 2 — Supplementary Information 2. [file 41598_2021_81025_MOESM2_ESM.docx]

**Table S1.** Initial treatment regimens for 406 chemo naïve patients with *Mycobacterium avium* complex pulmonary disease.

| **Initial treatment regimen (n=406)** | **No. (%)** |
| --- | --- |
| RFP+EB+CAM+FQ | 12 (3.0) |
| RFP+EB+CAM | 280 (69.0) |
| EB+CAM+FQ | 18 (4.4) |
| RFP+CAM+FQ | 25 (6.2) |
| EB+CAM | 23 (5.7) |
| RFP+CAM | 15 (3.7) |
| CAM+FQ | 21 (5.2) |
| CAM | 12 (3.0) |

RFP, rifampicin; EB, ethambutol; CAM, clarithromycin; FQ, fluoroquinolones.

**Table S2.** Treatment regimens for *Mycobacterium avium* complex pulmonary disease in this study.

| **Firstline treatment regimen (n=295)** | **No. (%)** |
| --- | --- |
| **・Standard regimen** |  |
| RFP+EB+CAM | 238 (80.7) |
| **・Alternative regimen** |  |
| EB+CAM+FQ | 14 (4.7) |
| RFP+CAM+FQ | 22 (7.5) |
| EB+CAM | 21 (7.1) |
| **Aminoglycoside use (n=85)** | **No. (%)** |
| AMK ->SM | 34 (40.0) |
| AMK -> KM | 27 (31.8) |
| SM | 17 (20.0) |
| KM | 8 (9.4) |

RFP, rifampicin; EB, ethambutol; CAM, clarithromycin; FQ, fluoroquinolones; AMK, amikacin; SM, streptomycin; KM, kanamycin.

**Table S3. Analysis of factors influencing choice of alternative regimens.**

| **Total (n=57)** |  |
| --- | --- |
| **Complications** | **Description** |
| Use of azole antifungal drugs (Voriconazole, n=1)) | Drug interaction with RFP. |
| Chronic thyroiditis (n=1) | Risk of hypo-thyroidism aggravation by RFP. |
| Systemic steroid use (n=3) | Drug interaction with RFP. |
| Digestive disease (n=1) | Avoided the use of RFP. |
| Chronic viral hepatitis (Type B, n=1; Type C, n=1) | Risk of augmented liver injury. |
| Chronic heart failure (n=1) | Drug interaction with RFP. |
| Ophthalmological disease (cataract, n=2, glaucoma, n=1) | Augmented risk of ophthalmological side effects by EB. |
| Poorly controlled diabetes mellitus (n=2) | Augmented risk of ophthalmological side effects by EB. |
| Alcoholic chronic hepatitis (n=1) | Augmented risk of ophthalmological side effects by EB, and liver side effects by RFP. |
| **Others** |  |
| Previous use of FQs with acceptable tolerance (n=4) |  |
| Suspicious of concomitant bacterial infection (Co-infection of P. aeruginosa (n=2). Concomitant pneumonia (n=3).) |  |
| Older age (n=5) | 2 drug EB+CAM regimen was selected. |
| Low body weight (n=3) | 2 drug EB+CAM regimen was selected. |
| Patient’s refusal to use 3 drug regimens due to concerns about side effects. (n=6) | 2 drug EB+CAM regimen was selected. |
| Unclear (n=19) |  |

**Table S4. Description of attending pulmonary physicians of patients with *Mycobacterium avium* complex pulmonary disease before and after statistical matching by propensity score.**

|  |  |  | **Statistical matching** | |
| --- | --- | --- | --- | --- |
|  | **Standard regimen (n=238)** | **Alternative regimen (n=57)** | **Matched**  **Standard regimen (n=48)** | **Matched**  **Alternative regimen (n=48)** |
| **Attending physician** | **Doctors; no.1 (n=1), no.2 (n=19), no.3 (n=3), no.4 (n=2), no.5 (n=5), no.7 (n=1), no.8 (n=1), no.9 (n=10), no.10 (n=2), no.11 (n=19), no.12 (n=16), no.13 (n=7), no.14 (n=23), no.15 (n=6), no.16 (n=1), no.17 (n=2), no.19 (n=6), no.20 (n=1), no.21 (n=1), no.23 (n=2), no.24 (n=14), no.25 (n=4), no.26 (n=4), no.27 (n=2), no.28 (n=3), no.29 (n=27), no.30 (n=1), no.31 (n=7), no.32 (n=4), no.33 (n=16), no.34 (n=5), no.35 (n=6), no.36 (n=8), no.37 (n=7), no.38 (n=2)** | **Doctors; no.2 (n=2), no.6 (n=1), no.7 (n=1), no.9 (n=2), no.11 (n=6), no.13 (n=3), no.14 (n=4), no.18 (n=1), no.19 (n=1), no.21 (n=1), no.22 (n=1), no.23 (n=1), no.24 (n=2), no.25 (n=1), no.26 (n=5), no.28 (n=1), no.29 (n=5), no.33 (n=15), no.35 (n=1), no.36 (n=2), no.37 (n=1)** | **Doctors; no.2 (n=1), no.7 (n=1), no.9 (n=3), no.11 (n=2), no.13 (n=2), no.14 (n=2), no.19 (n=2), no.21 (n=1), no.23 (n=2), no.24 (n=3), no.26 (n=4), no.28 (n=1), no.29 (n=9), no.33 (n=11), no.35 (n=1), no.37 (n=3)** | **Doctors; no.2 (n=2), no.7 (n=1), no.9 (n=2), no.11 (n=6), no.13 (n=3), no.14 (n=4), no.19 (n=1), no.21 (n=1), no.23 (n=1), no.24 (n=2), no.25 (n=1), no.26 (n=4), no.28 (n=1), no.29 (n=4), no.33 (n=11), no.35 (n=1), no.36 (n=2), no.37 (n=1)** |

**TableS5.** Risk factors for treatment failure in patients with MAC-PD.

|  |  |  | **Univariate analysis** | | **Multivariate analysis** | |
| --- | --- | --- | --- | --- | --- | --- |
|  | **Successful completion**  **(n=188)** | **Treatment failure (n=107)** | **p-value** | **OR (95%CI)** | **p-value** | **Adjusted**  **OR (95%CI)** |
| **Characteristic** |  |  |  |  |  |  |
| Sex, male | 39 (20.7) | 31 (29.0) | 0.1134 | 1.56 (0.90-2.69) | 0.061 | 1.81 (0.97-3.38) |
| Age, years | 65.6 (+/- 10.4) | 66.5 (+/- 10.6) | 0.4499 | 1.01 (0.99-1.03) |  |  |
| BMI | 18.9 (+/- 2.7) | 18.3 (2.6) | 0.0197^*^ | 0.91 (0.84-1.00) | 0.2515 | 0.94 (0.85-1.04) |
| Underlying disease |  |  |  |  |  |  |
| COPD | 10 (5.3) | 5 (4.7) | 0.807 | 0.87 (0.29-2.62) |  |  |
| Old Tb | 8 (4.3) | 7 (6.5) | 0.3973 | 1.58 (0.55-4.47) |  |  |
| DM | 16 (8.5) | 11 (10.3) | 0.6148 | 1.23 (0.55-2.76) |  |  |
| CPA | 2 (1.1) | 1 (0.9) | 0.9148 | 0.88 (0.08-9.79) |  |  |
| CRP | 1.1 (2.2) | 1.7 (2.3) | 0.0253^*^ | 1.13 (1.01-1.25) |  |  |
| NB form | 154 (81.9) | 95 (88.8) | 0.1103 | 1.75 (0.86-3.54) | 0.0567 | 2.29 (0.98-5.38) |
| Cavity | 72 (38.3) | 55 (51.4) | 0.0064^*^ | 1.90 (1.20-3.02) | 0.0136^*^ | 2.04 (1.16-3.59) |
| *M. avium* | 102 (54.3) | 58 (54.2) | 0.9934 | 1.00 (0.62-1.61) |  |  |
| AFB stain positive | 66 (35.1) | 57 (53.3) | 0.0024^*^ | 2.11 (1.30-3.42) | 0.021^*^ | 1.86 (1.10-3.16) |
| Standard regimen | 162 (86.2) | 76 (71.0) | Ref | 1.00 |  |  |
| Alternative regimen | 26 (13.8) | 31 (29.0) | 0.0018^*^ | 2.54 (1.41-4.58) | 0.017^*^ | 2.16 (1.15-4.07) |
| Aminoglycoside use | 61 (32.4) | 24 (22.4) | 0.0646 | 0.60 (0.35-1.04) | 0.0865 | 0.59 (0.32-1.08) |
| Adjuvant surgery | 10 (5.3) | 0 (0.0) | 0.0003^*^ | 2.38 (1.49-3.80) | 0.9969 |  |

OR, odds ratio; CI, confidence interval; BMI, body mass index; COPD, chronic obstructive pulmonary disease; Tb, tuberculosis; DM, diabetes mellitus; CRP, C-reactive protein; NB, nodular bronchiectasis; AFB, acid-fast bacilli; FQ, fluoroquinolone; EC, ethambutol and clarithromycin.

Data represent n (%) or median (interquartile range).

**Table S6.** Risk factors for recurrence in patients with MAC-PD.

|  |  |  | **Univariate analysis** | |
| --- | --- | --- | --- | --- |
|  | **Without Recurrence (n=146)** | **Recurrence (n=42)** | **p-value** | **OR (95%CI)** |
| **Characteristic** |  |  |  |  |
| Sex, male | 27 (18.5) | 12 (28.6) | 0.1673 | 1.76 (0.80-3.88) |
| Age, years | 65.3 (+/- 10.1) | 66.3 (11.5) | 0.6002 | 1.01 (0.98-1.04) |
| BMI | 18.8 (+/- 2.60) | 18.9 (+/- 2.91) | 0.3957 | 0.99 (0.94-1.03) |
| Underlying disease |  |  |  |  |
| COPD | 9 (6.2) | 1 (2.4) | 0.2952 | 0.37 (0.05-3.02) |
| Old Tb | 6 (4.1) | 2 (4.8) | 0.8554 | 1.17 (0.23-6.00) |
| DM | 12 (8.2) | 4 (9.5) | 0.7919 | 1.18 (0.36-3.85) |
| CPA | 1 (0.7) | 1 (2.4) | 0.3896 | 3.54 (0.22-57.8) |
| CRP | 1.56 (+/- 2.24) | 0.8 (+/- 1.74) | 0.3095 | 0.91 (0.75-1.11) |
| NB form | 118 (80.8) | 36 (85.7) | 0.4582 | 0.70 (0.27-1.83) |
| Cavitation | 60 (41.1) | 12 (28.6) | 0.5533 | 1.24 (0.60-2.56) |
| *M. avium* | 78 (53.4) | 24 (57.1) | 0.6695 | 0.86 (0.43-1.72) |
| AFB stain positive | 55 (37.7) | 11 (26.2) | 0.1724 | 0.58 (0.27-1.26) |
| Standard regimen | 125 (85.6) | 37 (88.1) | Ref | 1.00 |
| Alternative regimen | 21 (14.4) | 5 (11.9) | 0.6772 | 0.80 (0.28-2.28) |
| Aminoglycoside use | 48 (32.9) | 13 (31.0) | 0.8139 | 0.92 (0.44-1.92) |
| Adjuvant surgery | 9 (6.2) | 1 (2.4) | 0.354 | 0.37 (0.05-3.02) |

OR, odds ratio; CI, confidence interval; BMI, body mass index; COPD, chronic obstructive pulmonary disease; Tb, tuberculosis; DM, diabetes mellitus; CRP, C-reactive protein; NB, nodular bronchiectasis; AFB, acid-fast bacilli; FQ, fluoroquinolone; EC, ethambutol and clarithromycin.

Data represent n (%) or median (interquartile range).

**Table S7.** Comparison of fluoroquinolone (FQ) minimum inhibitory concentration (MIC).

| Patient no. | LVFX MIC (µg/mL) | MFLX MIC (µg/mL) | STFX MIC (µg/mL) |
| --- | --- | --- | --- |
| 35 | >16 | 4 |  |
| 213 | >16 | >8 |  |
| 95 | 2 | 1 |  |
| 144 | 1 | >8 |  |
| 96 | 1 | 4 |  |
| 155 | 0.25 | 0.12 | <0.03 |
| 163 | 0.5 | 0.25 | <0.03 |
| 88 | 1 | 2 |  |
| 165 | <=0.5 | <=0.25 |  |
| 176 | 1 | 0.5 | 0.06 |
| 145 | 0.25 | 0.12 | 0.25 |
| 75 | 8 | 4 |  |
| 121 | >16 | >8 |  |
| 157 | 4 | 1 | 0.12 |
| 16 | 4 | 2 | 1 |
| 166 | 1 | 0.5 | 0.12 |
| 162 | 4 | 0.5 | 0.12 |
| 87 | 16 | 4 |  |
| 126 | 1 | 2 | 1 |
| 170 | 0.5 | 0.25 | 0.06 |

LVFX, levofloxacin; MOFX, moxifloxacin; STFX, sitafloxacin.

**Table S8.** Risk factors for CPA development.

|  |  |  | **Univariate analysis** | | **Multivariate analysis** | |
| --- | --- | --- | --- | --- | --- | --- |
|  | **Without CPA diagnosis (n=272)** | **Development of CPA (n=23)** | **p-value** | **OR (95%CI)** | **p-value** | **Adjusted OR (95%CI)** |
| **Characteristic** |  |  |  |  |  |  |
| Sex, male | 60 (22.1) | 10 (43.5) | 0.0294^*^ | 2.72 (1.14-6.51) | 0.1285 | 2.12 (0.80-5.58) |
| Age, years | 65.9 (+/- 10.4) | 65.4 (+/- 8.09) | 0.8093 | 1.004 (0.97-1.05)) | 0.1296 | 1.03 (0.99-1.06) |
| BMI | 18.7 (+/- 2.6) | 18.0 (+/- 3.2) | 0.2532 | 0.91 (0.77-1.10) | 0.237 | 0.94 (0.84-1.05) |
| Underlying disease |  |  |  |  |  |  |
| COPD | 12 (4.4) | 3 (13.0) | 0.0857 | 3.25 (0.85-12.47) |  |  |
| Old Tb | 12 (4.4) | 2 (8.7) | 0.4191 | 1.90 (0.40-8.97) |  |  |
| DM | 24 (8.8) | 3 (13.0) | 0.5034 | 1.55 (0.43-5.60) |  |  |
| CRP | 1.1 (+/- 2.0) | 3.3 (+/- 3.2) | <.0001^*^ | 0.76 (0.66-0.87) | 0.0007^*^ | 1.32 (1.14-1.56) |
| NB form | 234 (86.0) | 15 (65.2) | 0.0117^*^ | 0.30 (0.12-0.77) | 0.2263 | 0.50 (0.16-1.55) |
| Cavity | 110 (40.4) | 17 (73.9) | 0.0003^*^ | 2.38 (1.49-3.80) | 0.2091 | 2.04 (0.67-6.22) |
| *M. avium* | 149 (54.8) | 11 (47.8) | 0.5215 | 0.76 (0.32-1.77) |  |  |
| AFB stain positive | 110 (40.4) | 13 (56.5) | 0.1385 | 1.91 (0.81-4.52) |  |  |
| Standard regimen | 222 (81.6) | 16 (69.6) | Ref | 1.00 |  |  |
| Alternative regimen | 50 (18.4) | 7 (30.4) | 0.1661 | 1.94 (0.76-4.97) |  |  |
| Aminoglycoside use | 76 (27.9) | 9 (39.1) | 0.2592 | 1.66 (0.69-3.99) |  |  |

MAC-PD, *Mycobacterium avium* complex pulmonary disease; OR, odds ratio; CI, confidence interval; BMI, body mass index; COPD, chronic obstructive pulmonary disease; Tb, tuberculosis; DM, diabetes mellitus; CRP, C-reactive protein; NB, nodular bronchiectasis; FQ, fluoroquinolone; CAM, clarithromycin; CPA, chronic pulmonary aspergillosis; AFB, acid-fast bacilli.

Data represent n (%) or median (interquartile range).

**Table S9.** Risk factors for mortality related to MAC-PD progression.

|  |  |  | **Univariate analysis** | | **Multivariate analysis** | |
| --- | --- | --- | --- | --- | --- | --- |
|  | **Censored (n=247)** | **Death related to MAC-PD progression (n=30)** | **p-value** | **HR (95%CI)** | **p-value** | **Adjusted HR (95%CI)** |
| **Characteristic** |  |  |  |  |  |  |
| Sex, male | 48 (19.4) | 12 (40.0) | 0.0098^*^ | 2.77 (1.29-5.73) | 0.0075^*^ | 3.35 (1.40-7.86) |
| Age, years | 65.3 (+/- 10.6) | 69.9 (+/- 10.4) | 0.0013^*^ | 1.07 (1.03-1.12) |  |  |
| BMI | 18.9 (+/- 2.6) | 16.6 (+/- 2.6) | <.0001^*^ | 0.64 (0.53-0.77) | 0.0003^*^ | 0.71 (0.59-0.85) |
| Underlying disease |  |  |  |  |  |  |
| COPD | 6 (2.4) | 3 (10.0) | 0.1425 | 2.77 (0.66-7.87) |  |  |
| Old Tb | 6 (2.4) | 5 (16.7) | 0.0126^*^ | 4.23 (1.42-10.23) | 0.0409^*^ | 3.40 (1.05-13.06) |
| DM | 16 (6.5) | 5 (16.7) | 0.0491^*^ | 2.99 (1.00-7.25) |  |  |
| CPA | 1 (0.4) | 1 (3.3) | 0.0208^*^ | 41.5 (2.19-243.1) |  |  |
| CRP | 1.1 (+/- 2.1) | 2.3 (+/- 2.3) | 0.001^*^ | 1.23 (1.10-1.36) | 0.0023^*^ | 1.31 (1.09-1.56) |
| NB form | 212 (85.8) | 27 (90.0) | 0.7655 | 0.84 (0.20-2.39) |  |  |
| Cavity | 92 (37.2) | 24 (80.0) | <.0001^*^ | 6.80 (2.95-18.44) | 0.0015 | 4.32 (1.49-10.48) |
| *M. avium* | 143 (57.9) | 13 () | 0.0008^*^ | 0.28 (0.11-0.59) | 0.0081^*^ | 0.34 (0.14-0.76) |
| AFB stain positive | 93 (37.7) | 20 (66.7) | 0.0072^*^ | 2.73 (1.31-6.08) | 0.0731 | 2.05 (0.94-4.75) |
| Standard regimen | 201 (81.4) | 25 (83.3) | Ref | 1.00 |  |  |
| Alternative regimen | 46 (18.6) | 5 (16.7) | 0.7734 | 1.15 (0.48-3.41) |  |  |
| Aminoglycoside use | 74 (30.0) | 6 (20.0) | 0.1226 | 0.51 (0.19-1.18) |  |  |
| Adjuvant surgery | 10 (4.0) | 0 (0.0) | 0.1528 |  | 0.182 |  |

MAC-PD, *Mycobacterium avium* complex pulmonary disease; HR, hazard ratio; CI, confidence interval; BMI, body mass index; COPD, chronic obstructive pulmonary disease; Tb, tuberculosis; DM, diabetes mellitus; CRP, C-reactive protein; NB, nodular bronchiectasis; FQ, fluoroquinolone; CAM, clarithromycin; CPA, chronic pulmonary aspergillosis; AFB, acid-fast bacilli.

Data represent n (%) or median (interquartile range).
